# Modeling Alzheimer’s disease through the integration of exposome, inflammasome, and connectome

**DOI:** 10.4103/NRR.NRR-D-25-00829

**Published:** 2025-09-29

**Authors:** Lorenzo Pini, Bruno P. Imbimbo, Manuela Allegra

**Affiliations:** Department of Neuroscience, University of Padova, Padova, Italy; Padova Neuroscience Center, University of Padova, Padova, Italy; Department of Research and Development, Chiesi Farmaceutici, Parma, Italy; Institute of Neuroscience, National Research Council (CNR), Padova, Italy

Over a century ago, the first clinical and neuropathological insights into major neurodegenerative diseases began to emerge: the description of Alzheimer’s disease (AD) by Alois Alzheimer in 1906, frontotemporal dementia by Arnold Pick in the same years, and Lewy bodies by Friedrich Lewy in 1912. These foundational studies laid the groundwork for the classification of what we now recognize as distinct neurodegenerative entities (Allali, 2024). Since then, decades of research have progressively deepened our understanding of the underlying pathophysiological mechanisms, drawing from in vitro models, animal studies, and human clinical observations. In recent years, these efforts have culminated in the development of the first pharmacological interventions targeting core pathological processes in AD. The approval of two anti-amyloid-β (Aβ) monoclonal antibodies, lecanemab and donanemab, in the United States, and more recently the approval of lecanemab in Europe, represents a milestone in translating biological discoveries into clinical applications. However, the clinical benefits observed thus far remain modest, and several critical challenges persist, notably the trade-off between limited efficacy and the risk of adverse effects. Moreover, for other conditions, such as frontotemporal dementia, the second most common cause of early-onset dementia, no effective treatments are currently available. This disparity, along with increasing scrutiny of the real-world impact of current AD therapies, underscores the need to integrate mechanistic paradigms with novel strategies that can help define new therapeutic priorities.

**Figure 1:**
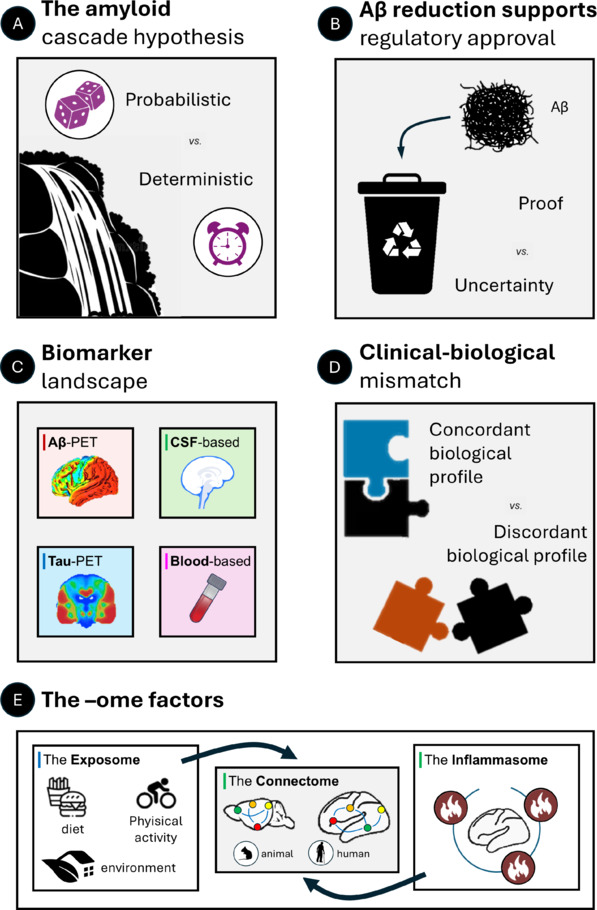
Overview of conceptual and translational challenges in AD research. (A) The amyloid cascade hypothesis: Emerging evidence supports a more heterogeneous and context-dependent role shaped by genetic, environmental, and stochastic factors. (B) Therapeutic efficacy: Is Aβ clearance a valid surrogate endpoint? Despite successful reduction of Aβ burden with several agents, the clinical benefits remain modest, prompting debate over the adequacy of amyloid reduction as a basis for regulatory approval. (C) Biomarker frameworks: Advances in fluid and imaging biomarkers have enhanced diagnostic precision and staging accuracy, but raise new questions about how best to interpret, integrate, and discretize (cut-off) markers of Aβ, tau, neurodegeneration, and comorbid pathologies. (D) Clinicopathological incongruence: A substantial proportion of individuals show either more advanced clinical symptoms than expected based on pathology, or vice versa, highlighting the importance of assessing co-pathologies and resilience mechanisms. (E) Inflammasome and exposome are emerging as potential factors influencing the pathophysiology of AD. The connectome could represent a common substrate in both animal models and patients for the integration of their effects on AD pathophysiology. Created with GIMP software. Aβ: Amyloid-β; AD: Alzheimer’s disease; CSF: cerebrospinal fluid; PET: positron emission tomography.

In this article, we pursue a twofold aim: (i) highlighting open questions in AD research and (ii) proposing that the interplay among different factors (exposome, inflammasome, and connectome) offers an integrated framework to reconcile inconsistencies in current AD models (**Figure 1**).

**Amyloid cascade: Deterministic or probabilistic?** A central issue in AD pathogenesis concerns the nature and role of Aβ. The hypothesis that Aβ is causative in AD emerged in the 1980s and has since dominated therapeutic research (amyloid cascade hypothesis; Hardy and Higgins, 1992). This model has posited that Aβ accumulation is the initiating and primary driver of a neurodegenerative process. However, this framework appears inconsistent with some empirical observations, such as the weak correlation between Aβ burden and cognitive decline. Further, previous clinical trials targeting Aβ have failed to demonstrate substantial therapeutic benefit (Zhang et al., 2024).

To address these inconsistencies, Frisoni and colleagues proposed a shift from a deterministic to a probabilistic AD model. This framework accounts for the heterogeneity observed in the disease and describes three AD variants (autosomal dominant, *APOE ε4*-related sporadic, and *APOE ε4*-unrelated sporadic) with progressively decreasing reliance on Aβ pathology and increasing influence from stochastic factors, including environmental exposures and lower-risk genetic contributors. Moreover, the relationship between Aβ and tau pathologies remains unclear, with tau pathology more consistently correlating with clinical progression. The interaction between Aβ and tau may be modulated by the *APOE ε4* genotype (Frisoni et al., 2022). These findings imply that the effects of Aβ accumulation are context-dependent and shaped by multiple biological processes, including neuroinflammation, cerebrovascular integrity, synaptic vulnerability, genetic susceptibility, and environmental factors.

**Assessing therapeutic efficacy: Is amyloid reduction enough?** A related issue concerns the use of amyloid clearance as a surrogate marker for treatment efficacy. Although several anti-amyloid drugs have successfully reduced brain amyloid levels, the associated clinical benefits have often been modest (Pini et al., 2024). This discrepancy raises an important question: is Aβ reduction alone a sufficient basis for the approval of anti-AD therapies?

Aisen and colleagues argue that reducing Aβ in asymptomatic individuals constitutes strong evidence of disease modification and should support regulatory approval. They also suggest that many earlier trial failures may not reflect flaws in the amyloid hypothesis itself, but limitations of past methodologies, such as the unavailability of Aβ cortical imaging or the inability to achieve substantial reductions in Aβ levels below baseline (Aisen et al., 2025). In contrast, Planche et al. (2025) adopt a more cautious position. They contend that Aβ clearance remains an unproven surrogate endpoint and that the evidence linking plaque reduction to meaningful clinical improvement is insufficient. As a result, they question whether Aβ-targeting therapies should be approved on the basis of biomarker outcomes alone (Planche et al., 2025).

**Rapidly evolving biomarker landscape:** Complicating these debates is the rapidly evolving landscape of biomarker definitions for amyloid, tau, neurodegeneration, and their respective cut-offs. Introduced in 2016, the ATN model shifted AD diagnosis from a clinical to a biological framework, classifying individuals by presence or absence of three core pathological processes: amyloid (A+/–), measured by amyloid positron emission tomography (PET) or Aβ_42_ in the cerebrospinal fluid (CSF); tau (T+/–), measured by tau PET or CSF phosphorylated tau (p-tau); and neurodegeneration (N+/–), assessed via structural magnetic resonance imaging, brain glucose metabolism, or CSF total tau. This model allows for a biology-based classification regardless of clinical status (e.g., an individual may be A+T+N+ but cognitively unimpaired).

Since 2018, refinement has improved the biomarker precision. CSF Aβ_42_ was replaced by the Aβ_42_/Aβ_40_ ratio to adjust for inter-individual variability. Plasma biomarkers emerged as novel alternatives. Tau markers advanced from CSF p-tau181 to p-tau217, which correlates better with amyloid plaque and tau tangle pathology and allows earlier detection of AD. Neurofilament light chain is gaining traction as a marker of neuronal injury. The 2018 NIA-AA research framework also acknowledged the need to expand beyond the original ATN classification, leading to the ATX(N) model. Here, “X” indicates additional biomarkers, such as inflammatory/immune activation (I), vascular brain injury (V), and α-synuclein pathology (S). Meanwhile, the “N” category was repositioned as a second-tier marker, informative for tracking disease progression but not essential for diagnosis. In the updated NIA-AA criteria, AD biomarkers are stratified into Core 1 and Core 2 categories (Jack et al., 2024). Core 1 includes Aβ (A) and early-changing phospho-tau markers (T1: p-tau217, p-tau181, p-tau231), which typically become abnormal concurrently with amyloid PET and well before tau PET. Core 2 includes late-changing tau biomarkers (T2: e.g., MTBR-tau243) and tau PET, which tend to become abnormal closer to clinical onset. This refined categorization reflects temporal dynamics in tau pathology and aims to improve staging accuracy.

These advancements have improved the accessibility and diagnostic precision of AD biomarkers. However, they have also introduced new complexities. Different biomarkers capture distinct aspects of the disease (structural, functional, or molecular), posing challenges in how to interpret and prioritize them in clinical and research settings. Similarly, the definition of cut-offs is problematic and relies on specific platform and analysis. The central question remains: which biomarkers best reflect disease-relevant biology, and how should they be weighted in evaluating treatment efficacy?

**AD pathophysiology and clinical profiles:** Always concordant? Perhaps the most puzzling challenge in recent years has been the observation of incongruence between biological markers and clinical symptoms in AD. Many individuals with high levels of Aβ or tau β remain cognitively unimpaired, while others with low levels of pathology exhibit substantial cognitive decline. This clinical–biological dissociation suggests that traditional neuropathological markers are insufficient to fully account for symptom onset and progression.

Recently, Pichet Binette et al. (2025) assessed the concordance between clinical and biological staging in two cohorts of participants. Individuals were classified into six clinical stages (ranging from cognitively normal to severe AD dementia) and four biological stages-based tau PET imaging. All participants included were Aβ-positive. In a first cohort, they reported clinical–biological congruence in 38% of cases. Notably, 51% of participants had a clinical stage more advanced than their biological stage, while 11% showed a biological stage more advanced than their clinical status (Pichet Binette et al., 2025). These results were replicated in an independent cohort, although congruent profiles were higher (56%), underscoring the need to measure non-AD biomarkers in patients whose cognitive impairment appears disproportionate to tau burden, as this may have implications for both diagnosis and prognosis.

**Emerging-omes in AD – exposome, inflammasome and connectome:** As evidence continues to accumulate, it is increasingly evident that the complexity of AD cannot be explained exclusively by Aβ pathology, tau deposition, or *APOE ε4* status. An increasingly relevant factor is the exposome, the cumulative lifetime environmental exposures. A second emerging player is the inflammasome, a multiprotein complex that mediates innate immune responses implicated in neuroinflammation processes. Among inflammasome complexes, NLRP3 has been consistently implicated in AD, particularly through its activation in microglia in response to Aβ and tau accumulation. Upon activation, NLRP3 triggers the release of pro-inflammatory cytokines and promotes a chronic neuroinflammatory state that exacerbates synaptic dysfunction and neurodegeneration. Neuroinflammation could damage vascular endothelial cells, leading to blood–brain barrier breakdown, which in turn amplifies microglial activation. This reciprocal interaction creates a feedback loop that may drive disease progression. These processes can be quantitatively measured at the macroscale using translocator protein PET for microglial activation and peripheral blood markers, such as interleukins. Notably, environmental exposures, central to the exposome, can modulate this activation, highlighting how the exposome and inflammasome influence each other. However, the precise mechanisms by which these factors interact with AD pathologies remain poorly understood.

The connectome, the comprehensive map of structural and functional brain connectivity, could represent a crucial translational bridge between exposome and inflammasome. Unlike traditional biomarkers that focus on static molecular deposits, connectomics captures dynamic brain alterations that can reflect both environmental exposures and immune-mediated neurodegeneration.

Animal studies provide a useful way to assess this relationship. For example, models of early-life environmental enrichment (characterized by enhanced sensory, motor, cognitive, and social stimulation) or environmental insults showed long-lasting signatures on brain network organization, even in genetically identical animals. In rodents, environmental enrichment has been shown to produce neuroplastic effects, mitigate neuroinflammation, and delay cognitive decline. Environmental enrichment may also attenuate Aβ and tau pathology in transgenic AD models. Recent findings suggest that these beneficial effects may be partially mediated by suppression of inflammasome activation, offering a direct mechanistic link between environmental exposures and immune modulation.

In humans, the concept of cognitive reserve represents the functional analogue of environmental enrichment. Shaped by education, occupational complexity, bilingualism, and sustained intellectual engagement, cognitive reserve is associated with reduced molecular pathology and may reflect long-term adaptations in large-scale brain networks. Neuroimaging-based connectomics can detect individual differences in brain network architecture that may relate to cognitive reserve, or more in general to lifetime exposures. Additionally, emerging evidence suggests that connectivity-based readouts could be sensitive to inflammasome-mediated damage, particularly in vulnerable brain hubs where neuroinflammation and metabolic stress often converge in AD. Elevated pro-inflammatory cytokines (e.g., interleukin-6) are linked to altered functional connectivity in both healthy and mild cognitive impairment individuals. Similarly, transient prenatal interleukin-6 elevation in mice has lasting effects on brain connectivity through excitatory synapses (Pini et al., 2023).

In this context, the integration of exposome, inflammasome, and connectome domains could represent a novel systems-level framework to understand AD pathophysiology. The exposome introduces variability and risk through lifelong environmental influences; the inflammasome acts as an immune sensor and amplifier of pathological cascades; the connectome captures brain network integrity.

Rather than treating these elements as isolated tools, this integrated framework highlights their dynamic interplay as essential to capturing AD pathophysiology. By linking environmental, immune, and network-level processes, the model complements traditional biomarker approaches and helps bridge preclinical and clinical research, offering a bidirectional translational model.

**Future directions:** In conclusion, identifying patterns of circuit dysfunction and mapping connectivity signatures that reflect both classical AD hallmarks and factors not directly related to the core pathophysiology may help align preclinical models more closely with human brain function. A critical limitation remains: there is currently no consensus on normative or pathological thresholds for brain connectivity. Similarly, current challenges remain in reliably measuring exposome variables. Establishing such benchmarks is essential to integrate connectivity metrics into diagnostic and prognostic workflows, bringing them on par with established biomarkers in the era of precision medicine. Finally, to better understand the full complexity of AD, we should also consider the interactions of the connectome with other “-omes,” like the epigenome, transcriptome, proteome, and microbiome. While these “-omes” themselves are not pathology nor the pathogenic mechanism, they serve as important research tools for investigating neurodegenerative processes and for developing novel approaches.
